# The impact of diagnosis on health-related quality of life in people with coeliac disease: a UK population-based longitudinal perspective

**DOI:** 10.1186/s12876-019-0980-6

**Published:** 2019-05-02

**Authors:** Mara Violato, Alastair Gray

**Affiliations:** Health Economics Research Centre, Nuffield Department of Population Health, University of Oxford, Richard Doll Building, Old Road Campus, Headington, Oxford, OX3 7LF UK

**Keywords:** Coeliac disease, Quality of life, Health economics

## Abstract

**Background:**

Before diagnosis, people with coeliac disease suffer reduced quality of life, which improves substantially after the disease has been diagnosed. Delayed diagnosis is common. The aim of this study was to assess changes over time in prevalence of coeliac disease symptoms/associated medical conditions, time to diagnosis, quality of life and its determinants before and after diagnosis in the United Kingdom.

**Methods:**

A postal questionnaire was designed in 2015 and sent to 4000 individuals with diagnosed coeliac disease, requesting information on respondents’ socio-demographic and clinical characteristics, and their quality of life pre- and post-diagnosis using the EQ-5D instrument. Data were analysed and compared with results from a survey conducted in 2006 using descriptive analyses, univariate and multivariable regression methods.

**Results:**

The survey response rate was 40%. Sixty-five percent of respondents reported at least 4 symptoms pre-diagnosis, a significant reduction by 13 percentage points (95% CI: -16.9, − 9.4; *p*-value: < 0.001) compared to 2006. Pre-diagnosis mean duration of symptoms was 12.8 years (SD: 15.3), a non-significant reduction of 0.6 years (95% CI: -2, 0.8; *p*-value: 0.426) compared to 2006. There was a significant improvement of 0.20 (95% CI: 0.18, 0.22; p-value: < 0.001) in quality of life from pre- (0.65) to post-diagnosis (0.85). Pre-diagnosis values were significantly higher by 0.09 (95% CI: 0.06, 0.12; *p*-value: < 0.001) than in 2006. Number of symptoms and low income were associated with decreased quality of life.

**Conclusions:**

Undiagnosed coeliac disease is associated with a substantial decrement in quality of life. Time to diagnosis has not significantly shortened over the decade 2006–2015, but symptoms are less severe when diagnosis occurs. Harmonising clinical guidelines for intensified active case finding will help improve quality of life of people with coeliac disease.

**Electronic supplementary material:**

The online version of this article (10.1186/s12876-019-0980-6) contains supplementary material, which is available to authorized users.

## Background

Coeliac disease (CD) is an inherited chronic autoimmune disorder triggered by ingestion of gluten [1], with prevalence typically 1–2% in international studies [2]. Diagnosis is usually prompted by a range of intestinal and extraintestinal symptoms, and by belonging to high-risk groups, such as having type 1 diabetes or first degree relatives with CD. Delayed diagnosis is common [1, 3–9]. The only currently available treatment is life-long adherence to a gluten-free (GF) diet, which patients may find socially restrictive and expensive [10–13]. In the UK, GF food available on prescription has been substantially reduced in recent years [14], with a consequent increase in costs to patients with CD [13].

Existing empirical evidence suggests that people with CD suffer reduced quality of life [3, 5–8, 15–18], especially prior to diagnosis. However, many studies rely on small sample sizes and often focus only on quality of life after diagnosis, usually in relation to adherence to a GF diet. Only a few studies have compared quality of life before and after diagnosis [6, 8], and, to our knowledge, none has investigated changes over time in quality of life before and after diagnosis using repeated surveys on large populations.

In this study we report on a large UK nationwide survey of people with CD, conducted in 2015, which largely replicated a previous survey we conducted in 2006 [6], providing a unique opportunity to assess changes over time in prevalence of CD symptoms/associated medical conditions, time to diagnosis, and quality of life before and after diagnosis.

## Methods

### Study population

A representative, geographically stratified, sample of 4000 individuals was drawn from the membership list of Coeliac UK, the leading charity supporting people with CD in the UK. Coeliac UK’s total membership comprises over 70,000 members, more than half the estimated UK population with CD [19]. Coeliac UK supports people who need to live gluten free, the majority of whom are diagnosed with CD or people with other medical conditions that benefit from a GF diet. Full details of the 2006 study population are reported elsewhere [6]. All members sampled were eligible for the study if they had a medically confirmed diagnosis of CD (self-report). In particular, 85.5% of the respondents were diagnosed through either a biopsy (53.2%; 842/1584) or blood test and biopsy (32.3%; 512/1584); 11% (175/1584) through blood test only; 3.1% indicated either other medical diagnostics (1.6%; 26/1584) such as enteroscopy/endoscopy, or a diagnosis by a medical professional (1.5%; 24/1584), such as specialist/consultant, without further specifying any diagnostics; finally, 0.3% (5/1584) of respondents reported they followed a GF diet and felt better, and were excluded from the analysis.

### Study design

All individuals in the 2015 survey were sent a questionnaire. The questionnaire design was informed by existing empirical literature on CD, current Coeliac UK priorities, and our prior experience in designing a similar survey for a comparable study in 2006. To ensure comparability of replies across the two survey times, we asked many of the same questions included in the 2006 survey, but also included additional questions. The questionnaire requested information on socio-demographic (e.g., gender, age, income) and clinical characteristics of respondents (e.g. type and duration of symptoms/CD associated medical conditions before and after diagnosis, time to and since diagnosis), and quality of life before and after diagnosis, using the EQ-5D-3L instrument [20]. Other data collected in the questionnaire included information on use of healthcare resources, out-of-pocket costs associated with CD before and after diagnosis, the impact of recent restrictions on NHS prescriptions of gluten-free (GF) products on affordability of a GF-diet, and the impact of CD on the day-to-day life of family and close friends of the person with CD – these data will be reported in a separate manuscript. No age restriction was placed on the sample, but for members aged under 18 years, a parent or guardian was asked to complete the questionnaire on their behalf.

The questionnaire was initially piloted with volunteers identified with the help of Coeliac UK, and revised in light of the feedback received. It was publicised in the Crossed Grain quarterly magazine sent to all Coeliac UK members, and mailed with a covering letter and a prepaid return envelope between the 11th and 18th September 2015. Responses were included in the analyses if received by the end of November 2015. The questionnaire is reproduced as [see Additional file 1]. Data were entered onto a database by three research assistants, and 10% of the sample was double-entered to check coding consistency. Inconsistencies were resolved by discussion and required amendments made to the data.

Ethics approval was granted by the Medical Science Inter-Divisional Research Ethics Committee of the University of Oxford, UK, reference number R36311_RE001.

### Statistical analysis

Descriptive statistics were used to characterize patient demographics and clinical details. Continuous variables were reported in terms of mean values, and/or mean differences of values before and after diagnosis, and categorical variables as numbers and percentages. Variability around mean values was measured by standard deviations, while precision around mean values and/or mean differences of key variables was reported using 95% confidence intervals. Missing data were not imputed. Statistical comparisons were made using t-tests and z-tests. Responses to the EQ-5D-3L questions were given a quality of life valuation using the UK specific valuation algorithm [20], and were compared with population norms derived from a national survey, age-standardized to the survey population [21].

Univariate and multivariable stepwise linear regressions were used to investigate predictors of quality of life before and after CD diagnosis for respondents to the 2015 survey. These analyses included sex, age at diagnosis, number and duration of symptoms/CD-associated medical conditions, number of comorbidities, family income, and region of residence and, for the after diagnosis period, also adherence to a GF diet, ease of access to GF food in shops, and change in eating out and travel patterns. Variables associated with quality of life before/after diagnosis with a *p*-value < 0.10, were included in the multivariable stepwise linear regression**.**

To assess differences over time in quality of life before and after diagnosis and their predictors, two further stepwise pooled regression analyses were conducted using both the 2006 and 2015 surveys data. The analyses were restricted to the sample of respondents who reported quality of life before and after diagnosis at each of the two survey time points. A *p*-value of < 0.05 was considered statistically significant, but we followed microeconometric practice in including regressors of theoretical and conventional importance even if not statistically significant (e.g. sex, age) [22].

Additional analyses were conducted on the 2015 key outcomes (duration of symptoms prior to diagnosis, quality of life before and after diagnosis) stratified by criteria of CD diagnosis, and in the sub-group of participants aged less than 18 years.

All statistical analyses were conducted using Stata 13 (StataCorp LP; College Station, TX).

## Results

### Characteristics of study participants

A response rate of 39.6% was achieved with 1584 completed and returned questionnaires (Table 1). Characteristics of the 2006 survey participants are reported here solely to permit comparison of findings over time. The majority of the 2015 respondents (75%) reported that they lived in England, 8% in Scotland, 4.5% in Wales, 2.5% in Northern Ireland, although 10% did not specify the country of residence. Twenty-seven percent of respondents were male and 73% female with an average age of 58 (SD: 21) and 55 (SD: 20) years, respectively. The average age of all respondents when surveyed was 56 (SD: 21) years, significantly older by 4 years (95% CI: 2.1, 5.4; *p*-value: < 0.001) than the average age in the 2006 survey. The average age of all respondents at diagnosis was 44 years (SD: 20), about 3 years older than in the 2006 survey (95% CI: 1.3, 4.6; p-value = 0.001); male respondents were diagnosed at a slightly older age (47 years) compared to women (43 years). Seventy-nine percent (1237/1559) of respondents were the only people in their family diagnosed with CD, while 21% (322/1559) had at least one other family member diagnosed with CD. The latter percentage was significantly higher by 14 percentage points (95% CI: 11, 16; *p*-value: < 0.001) than in the 2006 survey. Ninety-one percent (1427/1572) of respondents reported following a GF diet all the time. Information on this variable was not collected in the 2006 survey. Further characteristics are reported in Additional file 2: Table S1.

**Table 1:** Characteristics of study participants in 2015 and 2006 surveys

Variables	2015 survey	2006 survey
No. sampled	4000	2000
No. of completed and returned questionnaires	1584	788
Response rate	39.6%	39.4%
Sex – No. (%)		
Female	1151 (72.7)	559 (70.9)
Male	432 (27.3)	224 (28.4)
Average age at survey – Mean (SD)	56 (21)	52 (19)
Average age at diagnosis – Mean (SD)	44 (20)	41 (19)
Age at diagnosis (categories) – No (%)		
< 18	188 (12.8)	97 (12.4)
18–34	225 (15.3)	134 (17.2)
35–44	244 (16.6)	188 (24.1)
45–54	301 (20.5)	153 (19.6)
55–64	277 (18.8)	128 (16.4)
65+	236 (16)	80 (10.3)
Family members with diagnosed CD – No. (%)		
Respondent only person	1237 (79)	728 (93)
At least one other family member diagnosed	322 (21)	55 (7)
Following a GFD – No. (%)		
All the time	1427 (90.8)	N/A
Most of the time	131 (8.3)	N/A
Some/little/none of the time	14 (0.9)	N/A

### Type and duration of coeliac disease symptoms/associated medical conditions

Symptoms/CD-associated medical conditions prior to diagnosis are reported in Table 2 for both the 2015 and 2006 participants. The most common symptoms in the 2015 survey were abdominal pain/bloating (70.4%), diarrhoea (60.6%), chronic fatigue (59.3%), flatulence (50.4%), and anaemia (49.0%). These were also the most frequently reported symptoms in the 2006 survey, but the proportions reporting individual symptoms fell between the surveys, with particularly substantial reductions in the percentage of respondents reporting anaemia (mean difference: -15.9; 95% CI: -20, − 11.7; *p*-value: < 0.001) and diarrhoea (mean difference: -9.8; 95% CI: -13.8, − 5.8; p-value: < 0.001). Sixty-five percent (1021/1578) of the 2015 respondents reported at least 4 symptoms/CD-associated medical conditions, a significant reduction by 13 percentage points (95% CI: -16.9, − 9.4; *p*-value: < 0.001) compared to the 2006 survey. The percentage of 2015 respondents with asymptomatic CD prior to diagnosis was 2 percentage points higher than in 2006 (95% CI: 1, 3; *p*-value: 0.002).

**Table 2:** Frequency of reported symptoms/CD-associated medical conditions prior to diagnosis in 2015 and 2006 surveys

	Number (%) reporting symptoms/CD-associated medical conditions						Difference in % reporting symptoms/CD-associated medical conditions (2015–2006)		
	2015^a^			2006^b^					
	No.	%	(95% CI)	No.	%	(95% CI)	Difference in %	(95% CI)	*p*-value
Abdominal pain/bloating	1111	70.4	(68.2, 72.7)	556	70.7	(67.6, 73.9)	−0.3	(−4.2, 3.6)	0.867
Diarrhoea	956	60.6	(58.2, 63)	553	70.4	(67.2, 73.6)	−9.8	(−13.8, −5.8)	< 0.001
Chronic fatigue	935	59.3	(56.8, 61.7)	488	62.2	(58.8, 65.6)	−2.9	(−0.7, 1.3)	0.173
Flatulence	796	50.4	(48, 52.9)	368	46.9	(43.4, 50.4)	3.6	(−0.07, 7.8)	0.103
Anaemia	773	49.0	(46.5, 51.5)	509	64.8	(61.5, 68.2)	−15.9	(−20, −11.7)	< 0.001
Constipation	464	29.4	(27.2, 31.7)	207	26.3	(23.2, 29.4)	3.1	(−0.7, 6.9)	0.119
Mouth ulcer	451	28.6	(26.3, 30.8)	236	30.1	(26.8, 33.3)	−1.5	(−5.4, 2.4)	0.455
Joint pain	439	27.8	(25.6,30)	220	28.0	(24.8, 31.1)	−0.2	(−4.0, 3.7)	0.931
Headache	416	26.4	(24.2, 28.5)	232	29.6	(26.4, 32.8)	−3.2	(−7.1, 0.7)	0.101
Skin rash	350	22.2	(20.1, 24.2)	208	26.5	(23.4, 29.6)	−4.3	(−8, −0.6)	0.021
Depression	312	19.8	(17.8, 21.7)	185	23.6	(20.6, 26.6)	−3.8	(−7.4, −0.3)	0.032
Other symptoms^c^	248	15.7	(13.9, 17.5)	163	20.7	(17.9, 23.5)	−5.0	(−8.3, −1.6)	0.003
Osteoporosis	214	13.6	(11.9, 15.3)	92	11.7	(9.4, 13.9)	1.9	(−1, 4.7)	0.207
Ataxia	39	2.5	(1.7, 3.2)	39	5.0	(3.4, 6.5)	−2.5	(−4.2, −0.8)	0.001
Any symptom^c^	1535	97.3	(96.5, 98.1)	771	99.2	(98.6, 99.8)	−2.0	(−3, −1)	0.002
1–3 symptoms	514	32.6	(30.3, 34.9)	166	21.2	(18.3, 24)	11.4	(7.5, 14.9)	< 0.001
4+ symptoms	1021	64.7	(62.3, 67,1)	605	77.8	(74.1, 80)	−13.1	(− 16.9, −9.4)	< 0.001
No symptoms	43	2.7	(1.9, 3.5)	6	0.8	(0.2, 1.4)	2.0	(1, 3)	0.002

^a^ Sample size: 1578**;** ^b^ Sample size: 788; ^c^Including CD-associated medical conditions

The average duration of any symptoms/CD-associated medical conditions before diagnosis across the whole 2015 sample was 12.8 years (SD: 15.3), a non-significant reduction of less than 1 year (mean difference: -0.6; 95% CI: -2, 0.8; p-value: 0.426) compared to the 2006 survey [see Additional file 3: Table S2]. When restricting the sample to those diagnosed between 2012 and 2015 (33%, 442/1337), the mean duration of symptoms/CD-associated medical conditions reduced to 11 (SD: 14) years. There were few significant changes over time in the mean duration of specific symptoms/CD-associated medical conditions [see Additional file 3: Table S2].

Table 3 shows the frequency of reported symptoms/CD-associated medical conditions after diagnosis in the 2015 survey. Eighty-one percent of respondents reported experiencing symptoms/CD-associated medical conditions after diagnosis. The most common symptoms were abdominal pain/bloating (35%), diarrhoea (33%), joint pain (29%), and flatulence (28%). Thirty-two percent of respondents reported at least 4 symptoms/CD-associated medical conditions, and 19% reported no symptoms/CD-associated medical conditions after diagnosis. Comparable information was not collected in the 2006 Survey.

**Table 3:** Frequency of reported symptoms/CD-associated medical conditions after diagnosis – 2015 survey

Symptom/CD-associated medical condition	Number (%) reporting symptoms		
	No.	%	(95% CI)
Abdominal pain/bloating	559	35.4	(33.1, 37.8)
Diarrhoea	522	33.1	(30.8, 35.4)
Joint pain	451	28.6	(26.3, 30.8)
Flatulence	448	28.4	(26.2, 30.6)
Constipation	409	25.9	(23.8, 28.1)
Osteoporosis	333	21.1	(19.1, 23.1)
Chronic fatigue	321	20.3	(18.4, 22.3)
Headache	274	17.4	(15.5, 19.2)
Anaemia	228	14.5	(12.7, 16.2)
Skin rash	220	13.9	(12.2, 15.7)
Mouth ulcer	191	12.1	(10.5, 13.7)
Depression	180	11.4	(9.8, 13.0)
Other symptoms^a^	100	6.3	(5.1, 7.5)
Ataxia	30	1.9	(1.2, 2.6)
Any symptom^a^	1278	81.0	(79.1, 82.9)
1–3 symptoms	772	48.9	(46.5, 51.4)
4+ symptoms	506	32.1	(29.8, 34.4)
No symptom	300	19. 0	(17.1, 20.9)

^a^Including CD-associated medical conditions

### Quality of life before and after diagnosis

Table 4 reports the number and percentage of 2015 and 2006 respondents at different levels of each of the 5 questions comprising the EQ-5D-3L, before and after diagnosis, and the corresponding percentages at each level in the general population of England [21, 23], standardised to the age distribution of Coeliac UK respondents at the time of survey. Results are reported for respondents completing the EQ-5D questionnaires for both their pre-diagnosis and their current (at time of survey) health state (85% in 2015; 88% in 2006).

**Table 4:** Respondents’ self-reported health on EQ-5D before diagnosis and at the survey time, 2006 and 2015

EQ-5D Question	2015						2006					
	Level 1 No problem		Level 2 Some problems		Level 3 Severe problems		Level 1 No problem		Level 2 Some problems		Level 3 Severe problems	
	N	%	N	%	N	%	N	%	N	%	N	%
Mobility												
Before diagnosis	1126	83.4	209	15.5	15	1.1	524	75.2	159	22.8	14	2.0
After diagnosis	1169	86.6^**^	179	13.3^*^	2	0.2^***^	602	86.4^***^	93	13.3^***^	2	0.3^**^
*UK pop. norm*^*a*^		*73.1*		*26.7*		*0.2*		*78*		*22*		*0*
Self-care												
Before diagnosis	1295	95.9	48	3.6	7	0.5	648	93.0	35	5.0	14	2.0
After diagnosis	1305	96.7	40	3.0	5	0.4	673	96.6^**^	18	2.6^**^	6	0.9^*^
*UK pop. norm*^*a*^		90.0		6.5		0.5		*93*		*6*		*0*
Usual activities												
Before diagnosis	899	66.6	395	29.3	56	4.2	403	57.8	249	35.7	45	6.5
After diagnosis	1132	83.9^***^	198	14.7^***^	20	1.5^***^	574	82.4^***^	116	16.6^***^	7	1.0^***^
*UK pop. norm*^*a*^		75.9		21.5		2.6		*78*		*19*		*3*
Pain												
Before diagnosis	395	29.3	665	49.3	290	21.5	153	22.0	349	50.1	195	28.0
After diagnosis	857	63.5^***^	445	33.0^***^	48	3.6^***^	416	59.7^***^	252	36.2^***^	29	4.2^***^
*UK pop. norm*^*a*^		54.6		41.3		6.2		*58*		*37*		*4*
Anxiety/depression												
Before diagnosis	837	62.0	411	30.4	102	7.6	351	50.4	259	37.2	87	12.5
After diagnosis	1034	76.6^***^	285	21.1^***^	31	2.3^***^	518	74.3^***^	163	23.4^***^	16	2.3^***^
*UK pop. norm*^*a*^		*73.1*		*24.8*		*2.2*		*75*		*23*		*2*

^a^ UK population norms from Health Survey for England 2011 and 1996, respectively, standardised to age distribution of Coeliac UK survey respondents at the time of survey

Proportion of respondents reporting significantly different level of response at the time of survey compared to before diagnosis: ^***^ significant at the 1% level; ^**^significant at the 5% level; ^*^significant at the 10% level

In all but the self-care dimensions of the EQ-5D-3L, the proportion of respondents in the 2015 survey reporting no problems was significantly higher at the time of the survey (i.e. ‘after diagnosis’ rows) than before diagnosis (Table 4). This was particularly pronounced in the pain dimension, with 63.5% of respondents reporting themselves to have no problems at the time of the survey, compared to only 29.3% prior to diagnosis of CD. A similar pattern was observed in the 2006 survey, but the proportion of respondents reporting no problems before diagnosis was significantly higher in 2015 than in 2006 for all the EQ-5D domains [see Additional file 4: Table S3], especially in the anxiety/depression domain (mean difference: 12%; 95% CI: 0.07, 0.16; *p*-value: < 0.001).

The distribution of responses from a large general population survey in England [21], standardised to the age distribution of 2015 respondents at the time of survey, is also shown in Table 4 (see ‘UK pop. norm’ row): the proportion of respondents reporting no problems concerning ‘mobility’ or ‘self-care’ was higher than in the general population both before and after diagnosis, but substantially lower before diagnosis for the domains of ‘usual activities’, ‘pain’, and ‘anxiety’.

Placing valuations on the EQ-5D health states using the British “tariff” [20], the mean quality of life before diagnosis in the 2015 survey was 0.65 (where 0 = death and 1 = full health), and 0.85 at the time of the survey, indicating a highly statistically significant improvement of 0.20 (95% CI: 0.18, 0.22; *p*-value: < 0.001) (Table 5). Reported quality of life before diagnosis in the 2015 survey was significantly higher than in 2006 (mean difference: 0.09, 95% CI: 0.06, 0.12; p-value: < 0.001), but similar after diagnosis. When adjusting for differences between the 2006 and 2015 survey populations in a pooled analysis [see Additional file 5: Table S4], this improvement over time in pre-diagnosis quality of life increased from 0.09 to 0.20 (see coefficient ‘year 2015’). Post-diagnosis quality of life levels reported in our 2015 survey (Table 5) compare with an average quality of life in the general population of 0.80 in 2015 when age-standardised to age of respondents at time of survey response (see ‘UK population norm’ row, Table 5) [21].

**Table 5:** Respondents self-reported health on EQ-5D tariff and VAS before diagnosis and at the survey time

	2015		2006		2015–2006		
	Mean	95% CI	Mean	95% CI	Mean difference	95% CI	p-value
EQ-5D tariff							
Pre-diagnosis	0.65	(0.63, 0.67)	0.56	(0.54, 0.59)	0.09^***^	(0.06, 0.12)	< 0.001
Time of survey	0.85	(0.84, 0.86)	0.84	(0.82, 0.85)	0.01 s	(−0.01, 0.03)	0.210
Change	0.20^***^	(0.18, 0.22)	0.27^***^	(0.25, 0.30)	− 0.07^***^	(− 0.11, − 0.04)	< 0.001
*UK population norm*^*a*^	*0.80*	*(0.79,0.81)*	*0.82*	*(0.81, 0.83)*			
Visual Analogue Scale							
Pre-diagnosis	56%	(55, 57)	47%	(46, 49)	8%^***^	(6, 11)	< 0.001
Time of survey	80%	(79, 81)	80%	(79, 81)	0	n/a	n/a
Change	24%^***^	(23, 26)	33%^***^	(31, 35)	−9%^***^	(−11, −6)	< 0.001

^a^ Population Norm from Health Survey from England 2011 and 1996, respectively, adjusted to survey age at time of each survey

^***^ significant at the 1% level; ^**^significant at the 5% level; ^*^significant at the 10% level

On the Visual Analogue Scale (VAS – Table 5), 2015 respondents rated their health before diagnosis at 56% (0 = worst imaginable state, 100 = best imaginable state), and at 80% at the time of survey, a highly significant improvement of 24 percentage points (95% CI: 23, 26; *p*-value: < 0.001). In the 2006 survey, average pre-diagnosis health was 8 percentage points lower on the VAS than in 2015 (95% CI: 6, 11; *p*-value: < 0.001), with a similar post-diagnosis level.

### Factors associated with quality of life before and after diagnosis

Table 6 reports the results of a multivariable regression analysis of factors associated with quality of life before and after diagnosis in the 2015 survey (descriptive statistics and results of a 2015 univariate analysis are summarised in [see Additional file 2: Table S1] and [see Additional file 6: Table S5]). The number of symptoms/CD-associated medical conditions experienced by respondents was strongly related to quality of life both before and after diagnosis. Older age at diagnosis was associated with higher quality of life before diagnosis, with people aged more than 65 years at diagnosis having a pre-diagnosis quality of life higher by 0.17 (*p*-value: < 0.001) than people aged less than 18. After diagnosis the effect of age was non-significant. Lower quality of life was significantly associated with lower family income (<£20,000) both before and after diagnosis. After diagnosis, having more than three comorbidities, not having easy access to GF foods in the shops, and changes to travel patterns were significantly associated with decreased quality of life. Duration of symptoms/CD-associated medical conditions and poor adherence to a GF diet were also associated with lower quality of life, but these associations were only marginally significant (at the 10% level).

**Table 6:** Factors associated with quality of life before and after diagnosis – 2015 survey

	Before diagnosis^a^			After diagnosis^b^		
Covariates	Coeff.	95% CI	*p*-value	Coeff.	95% CI	*p*-value
Male	0.01	(−0.04, 0.05)	0.728	0.01	(−0.02,0.04)	0.400
Age at diagnosis						
Less than 18	reference			reference		
18–34	0.03	(−0.04, 0.10)	0.412	−0.01	(− 0.06, 0.03)	0.632
35–44	0.03	(−0.04, 0.10)	0.449	−0.02	(−0.07, 0.02)	0.333
45–54	0.08	(0.01, 0.14)	0.022	0.003	(−0.04, 0.05)	0.903
55–64	0.08	(0.01, 0.15)	0.022	−0.01	(−0.06, 0.03)	0.558
65+	0.17	(0.09, 0.24)	< 0.001	0.02	(−0.03, 0.06)	0.537
No. of symptoms ^c^						
None	reference			reference		
1–3 symptoms	−0.17	(−0.30, − 0.05)	0.007	− 0.05	(− 0.09, − 0.02)	0.003
4+ more symptoms	−0.40	(− 0.52, − 0.27)	< 0.001	−0.17	(− 0.22, − 0.13)	< 0.001
Max symptoms duration	–	–	–	−0.002	(− 0.004, 0.0004)	0.104
No. of comorbidities						
None	–	–	–	reference		
1 comorbidity	–	–	–	−0.02	(−0.05, 0.01)	0.158
2 comorbidities	–	–	–	− 0.03	(− 0.07, 0.003)	0.070
3+ comorbidities	–	–	–	− 0.08	(−0.12, − 0.04)	< 0.001
Income^d^ < £20,000	−0.06	(− 0.10, − 0.02)	0.008	−0.07	(− 0.10, − 0.04)	< 0.001
Adherence to a GF diet						
All of the time	–	–	–	reference		
Most of the time	–	–	–	− 0.04	(− 0.08, 0.003)	0.067
Some/little/none of the time	–	–	–	− 0.14	(−0.31, 0.03)	0.101
Access to GF products in shops						
Very easily	–	–	–	reference		
Fairly easily	–	–	–	−0.02	(−0.05, 0.004)	0.105
Not easily	–	–	–	−0.07	(− 0.11, − 0.02)	0.007
Travel patterns AD						
The same	–	–	–	reference		
More likely to travel	–	–	–	−0.08	(− 0.15, − 0.01)	0.028
Less likely to travel	–	–	–	−0.04	(− 0.07, 0.01)	0.002
Region^d^						
North East	−0.01	(−0.12, 0.10)	0.813	−0.04	(− 0.11, 0.03)	0.317
North West	0.03	(−0.05, 0.10)	0.471	−0.03	(−0.07, 0.02)	0.253
Yorkshire and The Humber	0.02	(−0.05, 0.09)	0.599	−0.01	(−0.05, 0.04)	0.808
East Midlands	0.003	(−0.07, 0.08)	0.927	-0.004	(−0.05, 0.04)	0.866
West Midlands	−0.05	(−0.14, 0.03)	0.207	−0.04	(− 0.10, 0.01)	0.095
East	−0.01	(−0.08, 0.06)	0.842	−0.01	(− 0.06, 0.03)	0.577
London	−0.09	(−0.18, 0.01)	0.079	−0.02	(− 0.08, 0.04)	0.511
South East	reference			reference		
South West	−0.01	(−0.08, 0.07)	0.872	−0.04	(−0.09, 0.01)	0.096
Northern Ireland	−0.04	(−0.15, 0.07)	0.494	−0.04	(− 0.11, 0.03)	0.319
Scotland	−0.03	(−0.11, 0.04)	0.422	−0.06	(− 0.11, − 0.01)	0.020
Wales	0.04	(−0.05, 0.14)	0.370	−0.001	(− 0.06, 0.06)	0.971

^a^ Sample size: 1076; ^b^ Sample size: 1007; ^c^ Including CD-associated medical conditions ^d^The income and geographical variables both refer to the time of the survey and, therefore, before diagnosis, they can only be considered as a proxy for socioeconomic status and geographical location

Pooled 2006–2015 data regression models [see Additional file 5: Table S4], which allowed assessing differences over time in quality of life before and after diagnosis and their predictors, also highlighted that being less likely to eat outside home after diagnosis compared with unchanged patterns of eating out was associated with significantly lower quality of life, but that this effect had lessened since the 2006 survey (coeff. on ‘Meals out AD’, category ‘Less likely’: - 0.07; 95% CI: -0.11; − 0.04; *p*-value: < 0.001). Being less likely to travel after diagnosis compared with unchanged patterns of travel was associated with significantly lower quality of life in both 2006 and 2015, and there was no significant difference in this result by the year of survey.

### Additional analysis

The additional analysis conducted on the 2015 key outcomes in the whole sample population, i.e. duration of symptoms prior to diagnosis, quality of life before and after diagnosis, indicates that those do not vary when stratified by CD diagnosis criteria [see Additional file 7: Table S6].

Further analyses conducted on the sub-group of participants aged less than 18 years indicate that the average duration of any symptoms/CD-associated medical conditions before diagnosis across the 2015 ‘< 18’ sample was 3.34 years (SD: 3.71), a non-significant increase of less than 1 year (mean difference: 0.6; 95% CI: -0.45, 1.58; *p*-value: 0.274) compared to the 2006 ‘< 18’ population (see Additional file 8: Table S7). Reported quality of life before diagnosis in the 2015 ‘< 18’ population was significantly higher than in 2006 (mean difference: 0.25, 95% CI: 0.14, 0.37; *p*-value: < 0.001), but was similar after diagnosis (i.e. time of survey). The mean quality of life before diagnosis in the 2015 ‘< 18’ population was 0.57 (where 0 = death and 1 = full health), and 0.88 at the time of the survey, indicating a highly statistically significant improvement of 0.31 (95% CI: 0.24, 0.37; p-value: < 0.001) [see Additional file 8: Table S7). On the Visual Analogue Scale (VAS – see Additional file 8: Table S7), the health before diagnosis of the 2015 ‘< 18’ population was rated at 46% (0 = worst imaginable state, 100 = best imaginable state), and at 83% at the time of survey, a highly significant improvement of 37 percentage points (95% CI: 33, 42; p-value: < 0.001). In the 2006 ‘< 18’ population, average pre-diagnosis health was 14 percentage points lower on the VAS than in 2015 (95% CI: 8, 20; p-value: < 0.001), with a similar post-diagnosis level. It has to be noted, however, that the sample sizes over which these sub-group analyses were conducted were small, i.e. 134 and 67 individuals for the 2015 and 2006 ‘< 18’ populations, respectively.

## Discussion

Patient reported outcome measures are increasingly recognised as valuable tools in assessing the burden of disease and the impact of interventions [2]. In this study we adopted a commonly used measure - the EQ-5D – in a relatively uncommon way, firstly by replicating a survey conducted 9 years previously, and secondly by asking respondents to rate their quality of life at the time of the survey but also retrospectively, prior to the diagnosis of their CD. A small number of previous studies have asked coeliac patients about their quality of life before and after diagnosis [5, 6, 8, 24], but we believe this is the first study to combine this approach with repeated surveys, providing important insights into changes in quality of life across the individual life-course but also over time at the population level.

Our findings confirm that, prior to diagnosis, the quality of life of people with CD is very substantially lower than in the general population. After diagnosis, however, quality of life improves substantially to levels similar to or better than in the general population. Similar results were found also in the sub-group analyses focusing on the ‘< 18’ population. This improvement following diagnosis has been found in a number of studies [5–8, 16, 17], and was evident in our 2006 and 2015 surveys, but our results show that quality of life pre-diagnosis was significantly better in 2015 than in 2006, suggesting that diagnosis is now occurring when the severity of symptoms/CD-associated medical conditions is less acute. However, we also found that the mean duration of symptoms/CD-associated medical conditions from onset to diagnosis was still 12.8 years in 2015, a non-significant reduction of less than one year compared with the mean delay of 13.3 years reported in the 2006 survey, despite increasing availability of highly sensitive serological tests, improved access to endoscopic services, and growing awareness of CD among healthcare professionals and patients [25]. In the sub-group analyses focusing on the ‘< 18’ population, time to diagnosis was 3.34 years in 2015, a non-significant increase of less than one year compared with the mean delay of 2.78 years reported in the 2006 survey, although the sample sizes on which these differences were estimated were small. Our estimated mean time to diagnosis of 11 years for the whole sample of patients diagnosed after 2012 compares with mean delays of 11.7 and 11 years reported in two North American studies [3, 7], conducted 10 and 15 years earlier than our 2015 study, respectively, and mean delays of 9.7 and 10 years in a Swedish and a Finnish study [8, 26], conducted respectively 6 years and 4 years before ours. Recent estimates from two further Finnish studies reported shorter delays [4, 5], although one reported that 32% of CD patients had a diagnostic delay of > 10 years [4], while a recent Swiss investigation reported a diagnostic delay of 7.3 years [9]. Long delays in diagnosis of CD therefore remain a widespread problem, particularly in light of the very substantial gains in quality of life associated with diagnosis.

As in 2006, gastrointestinal symptoms such as abdominal pain/bloating and diarrhoea remained the main clinical presentations before diagnosis in the 2015 study. However, the percentage of those reporting diarrhoea prior to diagnosis significantly reduced by 10 percentage points to 61% in 2015, in line with estimates ranging between 50 and 85% in previous studies across a variety of countries [3, 4, 7, 27, 28]. Abdominal pain/bloating and diarrhoea were experienced on average for 8 years in our 2015 survey before a CD diagnosis was made, in line with the results from a recent Finnish study [4], which reported gastrointestinal symptoms being associated with long delay to diagnosis. It has been suggested that increased awareness of CD may have improved the recognition of atypical symptoms, but that classical symptoms may have become milder and more prone to misdiagnosis, and some UK evidence supports this interpretation by reporting long delays in diagnosis of CD due to initial misdiagnosis of irritable bowel syndrome [29]. However, there could also be some recall bias, whereby common symptoms attributable to the disease may be overestimated compared with more atypical symptoms [4].

Persistent and/or recurrent symptoms in diagnosed and treated coeliac patients have been documented in previous studies [5, 18, 26], and are confirmed in this study: although the prevalence of symptoms declined markedly after diagnosis, 49% of respondents still reported 1–3 symptoms and 32% 4 or more symptoms, and we estimated the associated decrements in quality of life to be 0.05 and 0.17 respectively.

A small number of previous studies have examined the association between quality of life and income in people with CD after diagnosis. One small study reported no significant association in Brazil [30], while a US study reported an association between low income and poorer overall and CD-related health [31]. Our study is one of the first to demonstrate that respondents with lower incomes (<£20,000) had significantly lower quality of life than their higher-income counterparts both after and before diagnosis. One potential mechanism through which low income may negatively affect quality of life after CD diagnosis is through affordability of a GF diet [32]. In England, at the time of the 2015 survey, availability of GF food on the NHS prescription system was being reduced in various parts of the country, which may strengthen the quality of life-income association in future studies.

Long-term impairment in quality of life has often been attributed to a lack of strict compliance with a GF diet in existing studies [16, 24, 33]. Our 2015 results show that reduced quality of life after diagnosis was only weakly associated with poor adherence to a GF diet (although only small numbers were in this group), but strongly associated with poor access to GF products in shops. Limited availability of GF foods in shops, especially budget supermarkets which those on low incomes may be more likely to rely on, has been documented by previous UK and US studies [10, 11, 13].

Adhering to a life-long GF diet, however, may also negatively impact the lifestyle of individuals with CD by, for example, affecting the ability to dine out and travel, as several studies have indicated [3, 15, 34, 35]. We found that changes in travel patterns after compared with before diagnosis were significantly associated with lower quality of life in both our 2015 (Table 6) and pooled [see Additional file 5: Table S4] multivariable models, with no significant improvements in the 2006–2015 decade. However, our pooled analyses [see Additional file 5: Table S4] indicated that, although being less likely to eat out after than before diagnosis was associated with a significant reduction in quality of life in both surveys, the effect was smaller in 2015 (approximately a decrement of 0.02 compared to 0.07 in 2006: see Additional file 5: Table S4). These mixed results suggest that, while gluten-free products and better labelling have become more widespread in recent years [34], many people with CD still feel restricted in their ability to eat out safely when away from home or on holiday. In line with these results, when respondents to our 2015 survey were asked to rank a series of measures that government/society could take to help people with CD, the highest ranked option was “*Raise awareness of coeliac disease within society so that gluten-free meals can be easily available in canteens of schools and workplaces, cafés, pubs, restaurants”*.

The main strength and uniqueness of this study is that it provides the ability to examine changes over time in delays in CD diagnosis, age and symptoms/CD-associated medical conditions at diagnosis, and quality of life before and after diagnosis by means of two large samples of the UK population conducted 10 years apart using similar methodology. The newly published Tampere international task force on outcome measures in coeliac disease trials states that health-related quality of life should be considered a critical end point when assessing the value of a therapy or intervention to both patients and healthcare providers, but there is still very limited information in the published literature on quality of life in people with CD [2]. The current NICE clinical guideline on CD [36], for example, relies for some estimates of quality of life in its economic model on the findings of a small Argentinian study [37]. The evidence that we provide in this study is therefore timely and should robustly extend the limited existing evidence when modelling the cost-effectiveness of interventions in this area. The fact that we used the EQ-5D instrument, a widely adopted generic quality of life instrument which is specifically recommended by the National Institute of Health and Care Excellence in its recommended methods for technology appraisals [38], further strengthens the value of our estimates.

Our study also has some limitations. Our estimates of types and duration of symptoms and of quality of life prior to diagnosis were assessed retrospectively and are therefore susceptible to recall bias. This approach is, however, hard to avoid in the absence of very large long-term prospective studies with frequent data collection, and has been used widely in previous CD literature [3–8]. Our study aimed to include only respondents with clinically diagnosed CD. We used a comprehensive definition of medically diagnosed CD and respondents reported a range of diagnostic pathways; however, the percentages of respondents who indicated a diagnostics other than ‘biopsy’/ ‘biopsy plus blood test’ / ‘blood test’, or who only reported a diagnosis by a medical professional without specifying the diagnostics used, were only 1.6% (26/1584) and 1.5% (24/1584), respectively, and are therefore unlikely to bias our conclusions, even in the improbable event that those diagnoses were not truly medically confirmed. The additional analysis that we conducted on key outcomes stratified by criteria of CD diagnosis [see Additional file 7: Table S6] confirms that our results are robust to the type of CD diagnosis criteria used. We found no evidence that duration of symptoms or quality of life before or after diagnosis significantly varied across different diagnostic pathways. A question on CD diagnosis was not asked in the 2006 survey, but by virtue of the above robustness checks conducted in the 2015 survey, bias in the 2006 results, which are based on a similar population, is unlikely. Our study participants were drawn from the membership of Coeliac UK, which may not fully reflect the characteristics of all people with CD in the UK. Previous CD studies in a variety of countries have also recruited their study subjects from national patient-support organizations [3–6, 8, 15]. Our response rate of just under 40% is not atypical of postal questionnaire surveys, but again may have introduced some bias if respondents were systematically different from non-respondents. Additionally, given that the longitudinal perspective of our study relied on repeated cross-sectional rather than panel data, we cannot completely exclude that a small percentage of respondents may have participated in both surveys. However, the anonymous nature of the two surveys did not allow us to identify such potential individuals, and the fact that the 2015 sample was more than double in size the 2006 sample suggests that potential numbers may in fact be small. Finally, the current study design did not allow us to investigate quality of life stratified by type of CD (e.g. potential, refractory) but this could be an interesting and useful line of investigation in future research.

## Conclusion

In conclusion, our results show that people with CD typically experience a prolonged period prior to diagnosis with very substantially reduced quality of life, which usually returns to the normal levels of the general population after diagnosis and adherence to a gluten-free diet. We find little evidence that the time to diagnosis has shortened over the last 10 years, but some evidence that diagnosis is now occurring before quality of life falls as far as in the past. There is currently a lack of consensus on the evidence to support population screening for CD [39–42], and also between the various national and international guidelines that recommend case-finding in at-risk groups [36, 43–45]. But more active and better-informed case-finding should become a priority for reducing diagnostic delay and substantially improving the quality of life of people with coeliac disease.

## Additional files

Additional file 1: The impact of Coeliac Disease on Your Life: A Survey of Your Views. Original survey questionnaire. (DOCX 177 kb)

Additional file 2: **Table S1.** Other characteristics of study participants in 2015 and 2006 surveys. (DOCX 14 kb)

Additional file 3: **Table S2.** Duration of reported symptoms/CD-associated medical conditions prior to diagnosis in 2015 and 2006 surveys. (DOCX 14 kb)

Additional file 4: **Table S3.** Difference between 2015 and 2006 in respondents’ proportions of self-reported health on EQ-5D before diagnosis (retrospective) and at the time of the survey. (DOCX 13 kb)

Additional file 5: **Table S4.** Pooled regression analyses of 2015 and 2006 surveys before and after diagnosis of coeliac disease. (DOCX 15 kb)

Additional file 6: **Table S5.** Factors associated with quality of life before and after diagnosis – univariate analysis (2015 survey). (DOCX 16 kb)

Additional file 7: **Table S6.** Additional analysis on 2015 key outcome variables. (DOCX 12 kb)

Additional file 8: **Table S7.** Additional analysis on 2015 key outcome variables on the sub-group of individuals aged less than 18 years. (DOCX 12 kb)

## Abbreviations

CD: Coeliac disease
GF: Gluten-free
SD: Standard deviation
