# Supplementary material for: The impact of diagnosis on health-related quality of life in people with coeliac disease: a UK population-based longitudinal perspective
Source: BMC Gastroenterol. 2019 May 2;19:68. doi: 10.1186/s12876-019-0980-6 (PMC6498641; doi:10.1186/s12876-019-0980-6)
Supplement: Supplementary file 1 — The impact of Coeliac Disease on Your Life: A Survey of Your Views. Original survey questionnaire. (DOCX 177 kb) [file 12876_2019_980_MOESM1_ESM.docx]

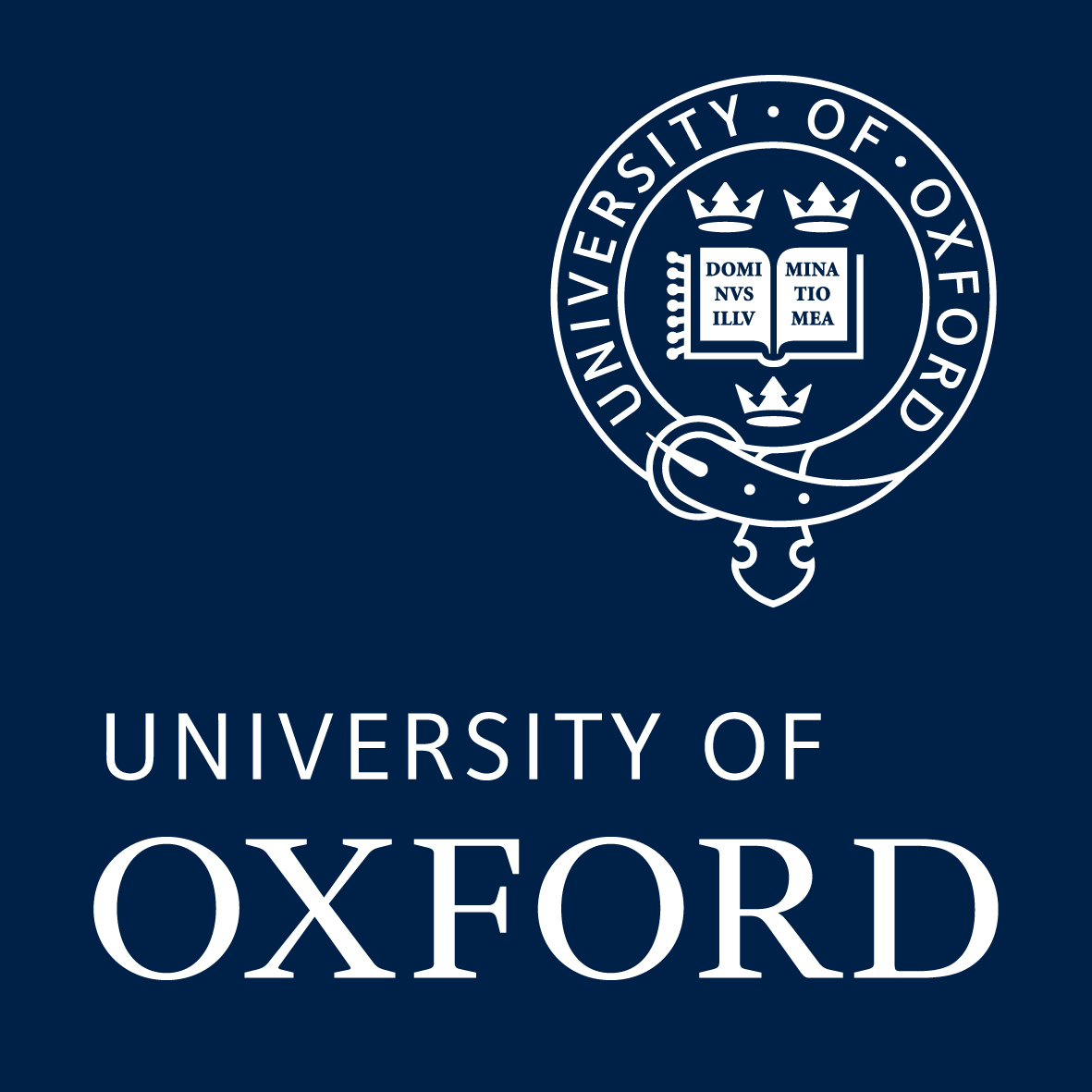

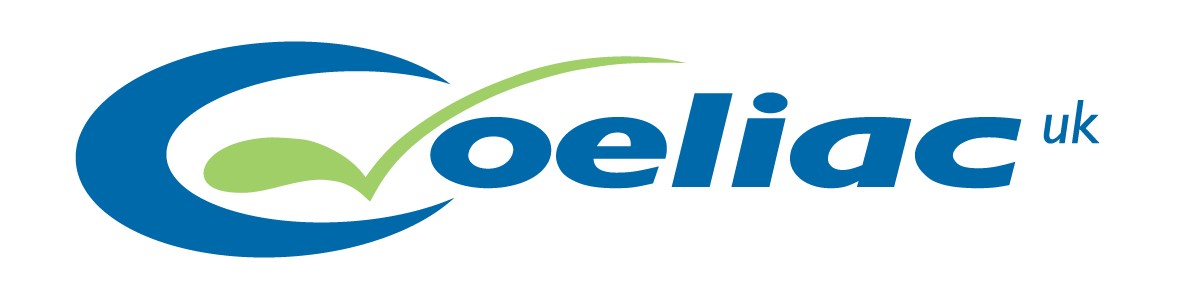


**The Impact of Coeliac Disease on Your Life:**

**A Survey of Your Views**

University of Oxford

(Health Economics Research Centre)

&

Coeliac UK

# Questionnaire – September 2015

| Please fill out the following questionnaire and return it to us in the stamped addressed envelope provided. Thank you for all your help. |
| --- |

| **Section A: Main Respondent Socio-Demographic and Clinical Details** |
| --- |

| **If you are filling out this questionnaire on behalf of a person with coeliac disease, for example a child, please tick the following box:** |
| --- |

1. Which gender do you associate yourself with? *Please tick as appropriate.*

Female

Male

1. Please state your postal code: _ _ _ _ _ _ _ _ _ _
2. Have you been diagnosed with coeliac disease by a medical professional? *Please tick as appropriate.*

Yes, by a blood test

Yes, by a biopsy

No, I adopted a gluten-free diet and felt better. *(If no, please return the questionnaire. Thank you for participating in the study)*

Other, please specify:

_ _ _ _ _ _ _ _ _ _

1. How old are you (in years)? _ _ _ _ _ _ _ _ _ _
2. How old were you (in years) when you were diagnosed with coeliac disease? _ _ _ _ _ _ _ _ _ _
3. Please state the number of family members currently living with you (excluding yourself).

_ _ _ _ _ _ _ _ _ _

1. Please state how many members of your family have been diagnosed with coeliac disease (excluding yourself): _ _ _ _ _ _ _ _ _ _
2. Have you been diagnosed with any other health condition(s), apart from coeliac disease? *Please tick as appropriate and specify the condition(s) which you have been diagnosed with.*

Yes I have been diagnosed with: _ _ _ _ _ _ _ _ _ _ _ _ _ _ _ _ _ _ _ _ _ _ _ _ _ _ _ _ _ _ _

No

1. What is your employment status? *Please tick all that apply.*

Employed

Job Seeker

Homemaker

Student

Retired

Other, please specify _ _ _ _ _ _ _ _ _

1. What is your socioeconomic background? (*If you are responding on behalf of a child please state the socioeconomic background of the head of the family* ) *Please tick as appropriate.*

Higher & intermediate managerial, administrative, professional occupations

*(E.g. director, accountant)*

Supervisory or clerical, junior managerial, administrative or professional

*(E.g. bank clerks, secretaries)*

Skilled manual workers

*(E.g. electricians, plumbers)*

Semi and unskilled manual workers

*(E.g. warehouse workers, construction)*

State pensioners or widows (no other earner), casual or minimum wage earners

1. To help assess the financial impact of coeliac disease, it is necessary to ask for the details of your family income. This information will be anonymised, and cannot be linked to individual respondents. It will be treated in the strictest confidence, if you wish to disclose it. What is your family gross annual income? *Please tick as appropriate.*

Less than £10, 000

£10, 000 - £20, 000

£20, 001 - £30, 000

£30, 001 - £40, 000

£40, 001 - £50, 000

£50, 001 - £60, 000

£60, 001 - £70, 000

More than £70, 000

I do not wish to say

1. What is the highest level of education you have completed? (*If you are responding on behalf of a child please state the highest level of education of the head of the family* ) *Please tick as appropriate.*

Secondary school

College (*further education*)

Undergraduate university degree

Postgraduate university degree

Other, please specify

_ _ _ _ _ _ _ _ _ _

1. What is your ethnicity? *Please tick as appropriate.*

White

Mixed

Indian

Pakistani or Bangladeshi

Black or Black British

Other, please specify:

_ _ _ _ _ _ _ _

1. Did you have any of the following symptoms **prior to diagnosis**? For each symptom that you had, whether continuous or intermittent, please tick the box next to it and indicate the **approximate** time that you had it for. If you had no symptoms please tick the ‘none of the above’ box.

| **Symptom** | **Tick if Experienced** | **Approximate Time Experienced** | |
| --- | --- | --- | --- |
|  |  | Months | Years |
| Diarrhoea |  |  |  |
| Constipation |  |  |  |
| Chronic Fatigue |  |  |  |
| Abdominal Pain /Bloating |  |  |  |
| Flatulence/excessive wind |  |  |  |
| Headaches |  |  |  |
| Joint pain |  |  |  |
| Osteoporosis |  |  |  |
| Skin Rashes |  |  |  |
| Mouth Ulcers |  |  |  |
| Anaemia |  |  |  |
| Depression |  |  |  |
| Ataxia |  |  |  |
| Other, please specify:  _ _ _ _ _ _ _ _ _ _ |  |  |  |
| None of the above |  |  |  |

1. Have you had any of the following symptoms **since diagnosis**? For each symptom that you have had, whether continuous or intermittent, please tick the box next to it and indicate the **approximate** time that you have had it for. If you have had no symptoms please tick the ‘none of the above’ box.

| **Symptom** | **Tick if Experienced** | **Approximate Time Experienced** | |
| --- | --- | --- | --- |
|  |  | Months | Years |
| Diarrhoea |  |  |  |
| Constipation |  |  |  |
| Chronic Fatigue |  |  |  |
| Abdominal Pain /Bloating |  |  |  |
| Flatulence/excessive wind |  |  |  |
| Headaches |  |  |  |
| Joint pain |  |  |  |
| Osteoporosis |  |  |  |
| Skin Rashes |  |  |  |
| Mouth Ulcers |  |  |  |
| Anaemia |  |  |  |
| Depression |  |  |  |
| Ataxia |  |  |  |
| Other, please specify:  _ _ _ _ _ _ _ _ _ _ |  |  |  |
| None of the above |  |  |  |

| **End of Section A.** |
| --- |

**Section B: Costs Associated with Coeliac Disease**

**Section B1: Costs Prior to Diagnosis**

| The following section will ask you about your costs when you had symptoms related to coeliac disease but had **not yet been diagnosed.** Please answer all questions for the **whole** of this time period. We realise that it may be difficult to give precise answers but it would be very useful to our study if you could give us your **best estimate.** |
| --- |

1. During the time that you had symptoms related to coeliac disease **but had not yet been diagnosed**, **approximately** how many times did you access the following NHS services **due to your symptoms**?

| **NHS health care service** | **Number of times before diagnosis** |
| --- | --- |
| GP consultation |  |
| Nurse consultation |  |
| Dietitian consultation |  |
| Referral to gastroenterologist |  |
| Referral to other consultants (*e.g. rheumatologist*) |  |
| Medical procedures (*e.g. enteroscopy*) |  |
| Routine medical examinations (*e.g. blood test*) |  |
| Hospitalisation (*e.g. inpatient care*) |  |

1. During the time that you had symptoms related to coeliac disease **but had not been diagnosed,** **approximately** how many days did you take off work/school/usual activities **due to your symptoms**?

_ _ _ _ **_** _ _ _ _ _

1. Please indicate **approximately** how much you spent (*including both consultation cost and any travel expenses*) on the following services and products, during the time that you had symptoms related to coeliac disease **but had not yet been diagnosed**. (*If unsure of precise costs, please give us your* ***best estimates***)

| **Service / Product** | **Cost (£) before diagnosis** |
| --- | --- |
| Private consultation(s) with gastroenterologists and/or other medical doctors | £ ……………………………………………. |
| Private consultation(s) with nutritional therapists / dietitians / homeopaths / and/or other therapists | £ ……………………………………………. |
| Private allergy testing | £ ……………………………………………. |
| Over-the-counter medications to alleviate symptoms | £ ……………………………………………. |
| Dietary products to help with experimentation on different diets to alleviate symptoms | £ ……………………………………………. |
| Nutritional supplements | £ ……………………………………………. |
| Books, DVDs, or videos on symptoms | £ ……………………………………………. |

**Section B2: Management of the Disease and Costs Following Diagnosis**

| The following section will ask you about your management of the disease and your costs in the time **since diagnosis** with coeliac disease. We realise that it may be difficult to give precise answers for the following questions, but it would be very useful to our study if you could give us your **best estimates**. |
| --- |

1. How well do you adhere to a gluten-free (GF) diet, aside from accidental consumption of gluten products? (*Please tick as appropriate*). I follow a GF-diet…

…all of the time

*…*most of the time

…some of the time

…a little of the time

…none of the time

1. **Since diagnosis,** **approximately** how many times have you accessed the following NHS services **due to your symptoms**?

| **NHS health care service** | **Number of times since diagnosis** |
| --- | --- |
| GP consultation |  |
| Nurse consultation |  |
| Dietitian consultation |  |
| Referral to gastroenterologist |  |
| Referral to other consultants (*e.g. rheumatologist*) |  |
| Medical procedures (*e.g. enteroscopy*) |  |
| Routine medical examinations (*e.g. blood test*) |  |
| Hospitalisation (*e.g. inpatient care*) |  |

1. Since you have been diagnosed, **approximately** how many days did you take off work/school/usual activities **due to your symptoms**?

_ _ _ _ **_** _ _ _ _ _

1. Have you had to change your professional career or training as a result of your diagnosis with coeliac disease? *Please tick as appropriate.*

Yes

No

Not applicable

1. Please indicate how much you spent (*including both consultation cost and any travel expenses*) on the following services and products **since diagnosis**. (*If unsure of precise costs, please give us your* ***best estimates***)

| **Service / Product** | **Cost (£) since diagnosis** |
| --- | --- |
| Private consultation(s) with gastroenterologists and/or other medical doctors | £ ……………………………………………. |
| Private consultation(s) with nutritional therapists / dietitians / homeopaths / and/or other therapists | £ ……………………………………………. |
| Private allergy testing | £ ……………………………………………. |
| Over-the-counter medications to alleviate symptoms | £ ……………………………………………. |
| Dietary products to help with experimentation on different diets to alleviate symptoms | £ ……………………………………………. |
| Nutritional supplements | £ ……………………………………………. |
| Books, DVDs, or videos on symptoms | £ ……………………………………………. |

1. Have you **ever** received gluten-free products on prescription? *Please tick as appropriate.*

Yes

No (*If no, please go to Q35*)

1. Are you **currently** receiving gluten-free products on prescription? *Please tick as appropriate.*

Yes (*If yes, please go to Q27*)

No

1. What was the main reason you stopped accessing gluten-free products on prescription? *Please tick as appropriate and then, please, go to Q35.*

Gluten-free products available on prescription are restricted/no longer available

I chose to as it is easier to get them from other sources

I chose to as I did not want prescription products

I chose to as I wanted a wider range of options

I chose to as I did not need to worry about the cost

I don't know

Other, please specify: _ _ _ _ **_** _ _ _ _ _

1. Have you **ever** experienced any restrictions to your prescriptions of gluten-free products? *Please tick as appropriate.*

Yes

No (*If no, please go to Q31*)

1. To what extent have you had your prescriptions of gluten-free items restricted at any time in the last few years? *Please tick as appropriate.*

| **Gluten-Free Products** | **Restricted** | **No longer available** | **Previously restricted/ unavailable, but is now available** | **No difference** |
| --- | --- | --- | --- | --- |
| Bread |  |  |  |  |
| Crackers |  |  |  |  |
| Pizza bases |  |  |  |  |
| Rolls |  |  |  |  |
| Pasta |  |  |  |  |
| Cakes/sweet biscuits |  |  |  |  |
| Flour |  |  |  |  |
| Other, please specify:  _ _ _ **_** _ _ _ _ _ |  |  |  |  |
|  |  |  |  |  |

1. Which of the following statements best describe your situation? *Please tick as appropriate.*

I cannot always afford to purchase the same amount and/or variety of gluten-free food that I was used to having on prescription

I am only just able to managing to purchase the same amount and/or variety of gluten-free food that I was used to having on prescription

I can still comfortably purchase the same amount and/or variety of gluten-free food that I was used to having on prescription

I no longer follow a strict gluten-free diet

1. If you have experienced restrictions on NHS prescriptions, please indicate **approximately** how much your **weekly** shopping bill for gluten-free products has changed due to those restrictions. *Please tick as appropriate.*

Has increased by less than £10 a week

Has increased by £10-£20 a week

Has increased by over £20 a week

Has stayed the same

1. **Approximately** how many different prescription items do you receive per month? *Please note that one item is one type of gluten-free product, regardless of how many packages you receive.*

_ _ _ _ **_** _ _ _ _ _

1. **Approximately** how many prescribed packs of gluten-free products (including loaves of bread) do you receive per month?

_ _ _ _ **_** _ _ _ _ _

1. How do you pay for your prescription items? *Please tick as appropriate.*

Through a prescription pre-payment certificate/season ticket

Through a prescription charge per item (*Please go to Q35*)

I am exempt from charges (*Please go to Q35*)

1. If you use a prescription pre-payment certificate/season ticket, what was the main reason you obtained one? *Please tick as appropriate.*

I have it mainly for other conditions

I have it mainly for gluten-free foods

1. How easily can you access gluten-free products in shops? *Please tick as appropriate.*

Very easily

Fairly easily

Not easily

1. Where do you have access to gluten-free products apart from via prescription? *Please tick as many as appropriate.*

Supermarket

Health food shops

Online specialist retailers

Gluten-free food events

Local corner shop / convenience shop

Other, please specify

_ _ _ _ _ _ _ _ _ _

1. Could you please indicate **approximately**, how much your **total weekly** shopping bill for food has changed **as a result of being diagnosed** with coeliac disease? *Please tick as appropriate.*

Has increased by over £40 a week

Has increased by £20-£40 a week

Has increased by less than £20 a week

Has stayed the same

Has decreased by less than £20 a week

Has decreased by £20-£40 a week

Has decreased by over £40 a week

1. **Since diagnosis**, how have your eating patterns changed at school/university/work due to your condition? *Please tick as appropriate. (If you are not currently in full time education or in employment please go to Q40)*

More likely to eat meals prepared at home

Less likely to eat meals prepared at home

No change

1. **Since diagnosis**, by how much have the **average weekly** costs of eating at school/university/work changed due to your condition? *Please tick as appropriate, and indicate by how much.*

Increased by: £_ _ _ _ _ _ _ _ _ _ a week

Decreased by: £_ _ _ _ _ _ _ _ _ _ a week

Stayed the same

I never purchase food at school/ university/work

1. **Since diagnosis**, are you more or less likely to eat a meal outside home (during evenings, on weekends and on holiday) because of your condition? *Please tick as appropriate.*

More likely

Less likely

The same

1. **Since diagnosis** how have the **average yearly** costs of eating a meal outside home (during evenings, on weekends and on holiday) changed because of your condition? *(Please tick the box corresponding to the choice that applies and indicate* ***your best estimate*** *of the amount by which they have changed).*

| **Change** | **Tick Box** | **Approximate Cost** |
| --- | --- | --- |
| They have increased |  | £ |
| They have decreased |  | £ |
| The have stayed the same |  | n/a |

1. **Since diagnosis** are you more or less likely to travel for studies/business/leisure because of your condition? *Please tick as appropriate.*

More likely

Less likely

The same

1. **Since diagnosis**, have you incurred any of the following household costs because of your condition? *Please tick as appropriate and indicate* ***approximately*** *how much the cost of each was.*

Buy new toaster(s) for: ­­­£_ _ _ _ _ _ _ _ _ _

Buy new freezer(s) for: ­­­£_ _ _ _ _ _ _ _ _ _

Buy bread maker for: ­­­£_ _ _ _ _ _ _ _ _ _

Buy new chopping boards for­­­: £_ _ _ _ _ _ _ _ _ _

Buy new cooking utensils for: £_ _ _ _ _ _ _ _ _ _

Other, please specify and state cost:

_ _ _ _ _ _ _ _ _ _ _ _ _ _ _ _ _ _ _ _ _ _ for £_ _ _ _ _ _ _ _ _ _

1. What measures do you feel the government/ society needs to take to help you better deal with coeliac disease and improve your wellbeing and the wellbeing of your family/friends? *Please rank with numbers 1-8 the following options, i.e. 1 the most important and 8 the least important.*

Raise awareness of coeliac disease within society so that gluten-free meals can be easily available in canteens of schools and workplaces, cafés, pubs, restaurants.

Increase the amount and range of gluten free products in local supermarkets.

Introduce mass-screening for coeliac disease so that the disease is detected and

managed without delays.

Improve health care for people with coeliac disease after diagnosis, so that management of the disease is regularly monitored.

Provide social support groups for people with coeliac disease and their families to better manage the condition.

Make sure that a gluten-free staple diet is available on NHS prescription.

Allow gluten free savoury snacks, cakes and biscuits to be/ remain available on

NHS prescription.

Fund further medical research in prevention and treatment of coeliac disease

If you have any other comments on Section B, please state them in the space provided below:

_ _ _ _ _ _ _ _ _ _ _ _ _ _ _ _ _ _ _ _ _ _ _ _ _ _ _ _ _ _ _ _ _ _ _ _ _ _ _ _ _ _ _ _ _ _ _ _ _ _ _ _ _ _ _ _ _ _ _ _ _ _ _ _ _ _ _ _ _ _ _ _ _ _ _ _ _ _ _ _ _ _ _ _ _ _ _ _ _ _ _ _ _ _ _ _ _ _ _ _ _ _ _ _ _ _ _ _ _ _ _ _ _ _ _ _ _ _ _ _ _ _ _ _ _ _ _ _ _ _ _ _ _ _ _ _ _ _ _ _ _ _ _ _ _ _ _ _ _ _ _ _ _ _ _ _

**End of Section B**

| **Section C: Quality of Life** |
| --- |

*This section asks you questions about your quality of life. Please indicate which statements best describe your health state prior to diagnosis by placing a tick in* ***one*** *of the three options for each question in column 1. Then repeat this process for your current health state by placing a tick in* ***one*** *of the three options for each question in column 2.*

| **Health State** | **Column 1: Before Diagnosis** | **Column2: Current**  **State** |
| --- | --- | --- |
|  |  | |
| **Question 1: Mobility**  I had/have no problems in walking about  I had/have some problems in walking about  I was/am confined to bed |  |  |
| **Question 2: Self-care**  I had/have no problems with self-care  I had/have some problems with washing or dressing myself  I was/am unable to wash and dress myself |  |  |
| **Question 3: Usual activities**  I had/have no problem in performing my usual activities *(e.g. work, study, housework, leisure activity)*  I had/have some problems in performing my usual activities  I was/am unable to perform my usual activities |  |  |
| **Question 4: Pain/Discomfort**  I had/have no pain or discomfort  I had/have moderate pain or discomfort  I had/have extreme pain or discomfort |  |  |
| **Question 5: Anxiety/Depression**  I was/am not anxious or depressed  I was/am moderately anxious or depressed  I was/am extremely anxious or depressed |  |  |

To help people say how good or bad a health state is we have drawn two scales (rather like thermometers) on which the best state you can imagine is marked by 100 and the worst state is marked by 0.

We would like you to indicate on the left hand scale (Scale 1) how good or bad your own health was, in your opinion, **prior to your diagnosis**. Please do this by drawing a **single line** from Box 1 to whichever point on Scale 1 indicates how your health state was.

We would then like you to indicate on the right hand scale (Scale 2) how good or bad your own health is, in your opinion, **currently**. Please do this by drawing a **single line** from Box 2 to whichever point on Scale 2 indicates how your health state is.

**Scale 1 Scale 2**

**100**

**0**

**90**

**70**

**60**

**50**

**40**

**20**

**30**

**80**

**10**

**100**

**0**

**90**

**70**

**60**

**50**

**40**

**20**

**30**

**80**

**10**

Best Imaginable Health State

Worst Imaginable Health State

**BOX 1**

**Health State Before Diagnosis**

**BOX 2**

**Current Health State**

| **End of Section C** |
| --- |

| **Section D: Impact of coeliac disease on family and friends** |
| --- |

| The aim of this section is to try to capture the impact that coeliac disease may have on the people close to you. The aim is to raise awareness of the influence that the disease may have beyond the individual diagnosed with it and to help identify areas where support to people with coeliac disease and their families/friends may be offered. Ideally the section should be completed by a close family member, partner or friend spending a fair amount of time with you. If that is not possible you may attempt to answer on their behalf. |
| --- |

1. What is your relation to the person with coeliac disease? *Please tick as appropriate.*

Partner/Family member living with someone with coeliac disease

Partner/Family member not living with someone with coeliac disease

Close friend (spending a fair amount of leisure time with the main respondent)

I am the person with coeliac disease answering on behalf of my partner/family member/friend

Other, please specify:

_ _ _ _ _ _ _ _ _ _

1. As a close person to someone with coeliac disease, in what situation(s) do you feel limited because wanting to be sympathetic with your partner/relative/friend’s condition? *Please tick all that apply.*

| At the supermarket |  |
| --- | --- |
| At pubs, cafés, restaurants |  |
| At parties, social events |  |
| During travel/holiday |  |
| During home visits to relatives or friends |  |
| None of the above |  |
| Other (please, specify) |  |

1. As a close person to someone with coeliac disease, how often do you feel restricted in choice when wishing to eat out? *Please tick as appropriate.*

Never

Occasionally

Sometimes

Frequently

All the time

1. Since your partner/relative/friend has been diagnosed with coeliac disease, how worried are you about her/his health and wellbeing? *Please tick as appropriate.*

I am not worried

I am moderately worried

I am extremely worried

1. Would you find it useful to attend social support meetings for families of individuals with coeliac disease? *Please tick as appropriate.*

Yes No Not applicable

1. We would like now to know how you rate your own health today. This question asks about your quality of life. *By placing a tick in* ***one*** *of the three options for each question below, please indicate which statements best describe your own health state today.*

**Question 1: Mobility**

I have no problems in walking about

I have some problems in walking about

I am confined to bed

**Question 2: Self-Care**

I have no problems with self-care

I have some problems washing or dressing myself

I am unable to wash or dress myself

**Question 3: Usual Activities *(e.g. work, study, housework, family or leisure activities)***

I have no problems with performing my usual activities

I have some problems with performing my usual activities

I am unable to perform my usual activities

**Question 4: Pain / Discomfort**

I have no pain or discomfort

I have moderate pain or discomfort

I have extreme pain or discomfort

**Question 5: Anxiety / Depression**

I am not anxious or depressed

I am moderately anxious or depressed

I am extremely anxious or depressed

To help people say how good or bad a health state is, we have drawn a scale (rather like a thermometer) on which the best state you can imagine is marked 100 and the worst state you can imagine is marked 0.

We would like you to indicate on this scale how good or bad your own health is today, in your opinion. Please do this by drawing a line from the box below to whichever point on the scale indicates how good or bad your health state is today.

**Scale**

**100**

| **End of Section D.** |
| --- |

Best Imaginable Health State

**0**

**90**

**70**

**60**

**50**

**40**

**20**

**30**

**80**

**10**

**BOX**

**Current Health State**

Worst Imaginable Health State

Thank you for taking the time

to participate in our study.

Your experience is of great value.
