# Supplementary material for: The impact of diagnosis on health-related quality of life in people with coeliac disease: a UK population-based longitudinal perspective
Source: BMC Gastroenterol. 2019 May 2;19:68. doi: 10.1186/s12876-019-0980-6 (PMC6498641; doi:10.1186/s12876-019-0980-6)
Supplement: Supplementary file 2 — Table S1. Other characteristics of study participants in 2015 and 2006 surveys. (DOCX 14 kb) [file 12876_2019_980_MOESM2_ESM.docx]

**Additional file 2**

**Table S1 – Other characteristics of study participants in 2015 and 2006 surveys**

| **Variables** | **2015 survey** | **2006 survey** |
| --- | --- | --- |
| *Socioeconomic background^a^* | 601 (40.3) |  |
| Higher & intermediate managerial, administrative, professional occupations | 423 (28.3) | N/A |
| Supervisory or clerical, junior managerial, administrative or professional | 82 (5.5) | N/A |
| Skilled manual workers | 72 (4.8) | N/A |
| Semi and unskilled manual workers | 315 (21.1) | N/A |
| State pensioners or widows (no other earner), casual or minimum wage earners | 601 (40.3) | N/A |
| *Annual family income (gross)^a^* |  |  |
| < £20,000 | 434 (29.1) | N/A |
| ≥ £20,000 | 1059 (70.9) | N/A |
| *Highest educational status^a^* |  |  |
| Secondary school | 469 (30.2) | N/A |
| College (further education) | 454 (29.3) | N/A |
| Undergraduate university degree | 278 (17.9) | N/A |
| Postgraduate university degree | 219 (14.1) | N/A |
| Other | 132 (8.5) | N/A |
| *Max duration of symptoms^b^*  *before diagnosis* (years) | 12.8 (15.3) | 13.3 (16.1) |
| *Max duration of symptoms^b^*  *after diagnosis* (years) | 8.4 (8.9) | N/A |
| *Time since diagnosis^b^* (years) | 11.4 (11.9) | 10.6 (10.7) |
| *No. of comorbidities^a^* |  |  |
| None | 543 (34.8) | N/A |
| 1 comorbidity | 539 (34.6) | N/A |
| 2 comorbidities | 265 (17) | N/A |
| 3+ comorbidities | 212 (13.6) | N/A |
| *Access to GF products in shops^a^* |  |  |
| Very easily | 574 (36.9) | N/A |
| Fairly easily | 849 (54.5) | N/A |
| Not easily | 134 (8.6) | N/A |
| *Meals out after diagnosis^a^* |  |  |
| The same | 446 (28.6) | 227 (29.4) |
| More likely | 36 (2.3) | 24 (3.10) |
| Less likely | 1079 (69.1) | 522 (67.5) |
| *Travel patterns after diagnosis^a^* |  |  |
| The same | 965 ( 64.6) | 539 (69) |
| More likely to travel | 37 (2.5) | 27 (3.4) |
| Less Likely to travel | 492 (32.9) | 215 (27.5) |
| *Region^a^* |  |  |
| North East | 46 (3.2) | N/A |
| North West | 142 (10) | N/A |
| Yorkshire and The Humber | 149 (10.5) | N/A |
| East Midlands | 145 (10.2) | N/A |
| West Midlands | 97 (6.82) | N/A |
| East | 144 (10.13) | N/A |
| London | 73 (5.13) | N/A |
| South East | 243 (17.1) | N/A |
| South West | 144 (10.1) | N/A |
| Northern Ireland | 41 (2.9) | N/A |
| Scotland | 124 (8.7) | N/A |
| Wales | 74 (5.2) | N/A |

*^a^* Number (%); *^b^* Mean (Std. Dev.)
