# Supplementary material for: The impact of diagnosis on health-related quality of life in people with coeliac disease: a UK population-based longitudinal perspective
Source: BMC Gastroenterol. 2019 May 2;19:68. doi: 10.1186/s12876-019-0980-6 (PMC6498641; doi:10.1186/s12876-019-0980-6)
Supplement: Supplementary file 3 — Table S2. Duration of reported symptoms/CD-associated medical conditions prior to diagnosis in 2015 and 2006 surveys. (DOCX 14 kb) [file 12876_2019_980_MOESM3_ESM.docx]

**Additional file 3**

**Table S2– Duration of reported symptoms/CD-associated medical conditions prior to diagnosis in 2015 and 2006 surveys**

|  | **Duration of symptoms/CD-associated medical conditions** | | | | | | **Mean Difference duration of symptoms/CD-associated medical conditions**  **(2015-2006)** | | |
| --- | --- | --- | --- | --- | --- | --- | --- | --- | --- |
|  | **2015** | | | **2006** | | |  |  |  |
|  | **Mean**  **(years)** | **(95% CI)** | ***No. Obs.*** | **Mean**  **(years)** | **(95% CI)** | ***No. Obs.*** | **Mean Difference**  **(years)** | **(95% CI)** | **p-value** |
| **Any symptom^a^** | 12.8 | (11.9, 13.6) | *1337* | 13.3 | (12.2, 14.5) | *719* | -0.6 | (-2.0, 0.8) | 0.426 |
| **Abdominal pain/bloating** | 8.3 | (7.6, 9.1) | *907* | 7.9 | (6.8, 8.9) | *490* | 0.5 | (-0.8, 1.7) | 0.464 |
| **Diarrhoea** | 7.9 | (7.1, 8.7) | *806* | 6.9 | (5.9, 8.0) | *511* | 1.0 | (-0.3, 2.3) | 0.143 |
| **Chronic fatigue** | 6.6 | (5.9, 7.2) | *790* | 7.1 | (6.0, 8.2) | *436* | -0.6 | (-1.8, 0.7) | 0.372 |
| **Flatulence** | 10.0 | (9.0,11.1) | *620* | 9.6 | (8.1, 11.0) | *317* | 0.5 | (-1.3, 2.3) | 0.596 |
| **Anaemia** | 10.8 | (9.6, 12.0) | *566* | 11.6 | (10.1, 13.0) | *433* | -0.8 | (-2.6, 1.1) | 0.413 |
| **Constipation** | 11.8 | (10.3,13.3) | *367* | 12.6 | (10.3, 14.8) | *180* | -0.8 | (-3.4, 1.9) | 0.572 |
| **Mouth ulcer** | 11.7 | (10.1, 13.3) | *329* | 11.3 | (9.1, 13.4) | *208* | 0.4 | (-2.2, 3.0) | 0.754 |
| **Joint pain** | 9.2 | (8.0, 10.4) | *329* | 8.2 | (6.6, 9.8) | *187* | 0.9 | (-1.0, 2.9) | 0.352 |
| **Headache** | 13.2 | (11.5, 14.8) | *309* | 10.3 | (8.4, 12.3) | *203* | 2.8 | (0.2, 5.4) | 0.033 |
| **Skin rash** | 9.8 | (8.3, 11.2) | *261* | 10.0 | (7.9, 12) | *176* | -0.2 | (-2.7, 2.3) | 0.866 |
| **Depression** | 9.9 | (8.3, 11.4) | *227* | 9.3 | (7.4, 11.2) | *169* | 0.6 | (-1.9, 3.0) | 0.654 |
| **Other symptoms^a^** | 6.1 | (4.7, 7.6) | *177* | 5.7 | (4.1, 7.2) | *126* | 0.5 | (-1.7, 2.6) | 0.660 |
| **Osteoporosis** | 9.6 | (7.6, 11.5) | *128* | 7.9 | (4.7, 11.0) | *69* | 1.7 | (-1.8, 5.2) | 0.346 |
| **Ataxia** | 9.9 | (5.5, 14.2) | *28* | 6.1 | (1.8, 10.3) | *33* | 3.8 | (-2.2, 9.8) | 0.210 |

^a^Including CD-associated medical conditions
