# Supplementary material for: The impact of diagnosis on health-related quality of life in people with coeliac disease: a UK population-based longitudinal perspective
Source: BMC Gastroenterol. 2019 May 2;19:68. doi: 10.1186/s12876-019-0980-6 (PMC6498641; doi:10.1186/s12876-019-0980-6)
Supplement: Supplementary file 4 — Table S3. Difference between 2015 and 2006 in respondents’ proportions of self-reported health on EQ-5D before diagnosis (retrospective) and at the time of the survey. (DOCX 13 kb) [file 12876_2019_980_MOESM4_ESM.docx]

**Additional file 4**

**Table S3 - Difference between 2015 and 2006 in respondents’ proportions of self-reported health on EQ-5D before diagnosis (retrospective) and at the time of the survey**

| **EQ-5D Question** | **Difference in percentages**  **(2015 – 2006)** | | | | | |
| --- | --- | --- | --- | --- | --- | --- |
|  | **Level 1**  **No problem** | | **Level 2**  **Some problems** | | **Level 3**  **Severe Problems** | |
|  | Difference | (95% CI) | Difference | (95% CI) | Difference | (95% CI) |
| **Mobility** |  |  |  |  |  |  |
| Before diagnosis | 0.08^***^ | (0.04, 0.12) | -0.07^***^ | (-0.11, -0.04) | -0.01^*^ | (-0.02, 0.003) |
| After diagnosis | 0.002 | (-0.03, 0.03) | 0.00 | (-0.03, 0.03) | -0.001 | (-0.01, 0.003) |
|  |  |  |  |  |  |  |
| **Self-care** |  |  |  |  |  |  |
| Before diagnosis | 0.03^***^ | (0.01, 0.05) | -0.01 | (-0.03, 0.004) | -0.01^***^ | (-0.03, -0.004) |
| After diagnosis | 0.001 | (-0.02, 0.02) | 0.00 | (-0.01, 0.02) | -0.005 | (-0.01, 0.003) |
|  |  |  |  |  |  |  |
| **Usual activities** |  |  |  |  |  |  |
| Before diagnosis | 0.09^***^ | (0.04, 0.013) | -0.06^***^ | (-0.11, -0.02) | -0.02^**^ | (-0.04, -0.002) |
| After diagnosis | 0.01 | (-0.02, 0.05) | -0.02 | (-0.05, 0.01) | 0.005 | (-0.01, 0.015) |
|  |  |  |  |  |  |  |
| **Pain** |  |  |  |  |  |  |
| Before diagnosis | 0.07^***^ | (0.03, 0.11) | -0.01 | (-0.05, 0.04) | -0.06^***^ | (-0.10, -0.025) |
| After diagnosis | 0.04^*^ | (-0.01, 0.08) | -0.03 | (-0.08, 0.01) | -0.01 | (-0.02, 0.012) |
|  |  |  |  |  |  |  |
| **Anxiety/depression** |  |  |  |  |  |  |
| Before diagnosis | 0.12^***^ | (0.07, 0.16) | -0.07^***^ | (-0.11, -0.02) | -0.05^***^ | (-0.08, -0.021) |
| After diagnosis | 0.02 | (-0.02, 0.06) | -0.02 | (-0.06, 0.02) | 0.00001 | (-0.01, 0.014) |

^***^ significant at the 1% level; ^**^significant at the 5% level; ^*^significant at the 10% level
