# Supplementary material for: The impact of diagnosis on health-related quality of life in people with coeliac disease: a UK population-based longitudinal perspective
Source: BMC Gastroenterol. 2019 May 2;19:68. doi: 10.1186/s12876-019-0980-6 (PMC6498641; doi:10.1186/s12876-019-0980-6)
Supplement: Supplementary file 5 — Table S4. Pooled regression analyses of 2015 and 2006 surveys before and after diagnosis of coeliac disease. (DOCX 15 kb) [file 12876_2019_980_MOESM5_ESM.docx]

**Additional file 5**

**Table S4 – Pooled regression analyses of 2015 and 2006 surveys before and after diagnosis of coeliac disease**

|  | **Before diagnosis**^a^ | | | **After diagnosis**^b^ | | |
| --- | --- | --- | --- | --- | --- | --- |
| ***Covariates*** | **Coeff.** | **95% CI** | **p-value** | **Coeff.** | **95% CI** | **p-value** |
| *Male* | 0.02 | (-0.01, 0.06) | 0.145 | 0.04 | (0.02, 0.06) | <0.001 |
| *Year 2015* | 0.20 | (0.11, 0.28) | <0.001 | -0.01 | (-0.05, 0.03) | 0.511 |
| *Age at diagnosis* |  |  |  |  |  |  |
| <18 | *reference* |  |  | *reference* |  |  |
| 18-34 | 0.19 | (0.10, 0.28) | <0.001 | -0.02 | (-0.05, 0.02) | 0.389 |
| 35-44 | 0.18 | (0.10, 0.27) | <0.001 | -0.03 | (-0.07, 0.002) | 0.066 |
| 45-54 | 0.24 | (0.16, 0.33) | <0.001 | -0.03 | (-0.07, 0.01) | 0.100 |
| 55-64 | 0.31 | (0.21, 0.40) | <0.001 | -0.04 | (-0.08, -0.01) | 0.022 |
| 65+ | 0.34 | (0.24, 0.45) | <0.001 | -0.03 | (-0.07, 0.01) | 0.099 |
| *Age diag.* x *Year 2015* |  |  |  |  |  |  |
| <18 x Year 2015 | *reference* |  |  | -- | -- | -- |
| 18-34 x Year 2015 | -0.17 | (-0.28, -0.05) | 0.004 | -- | -- | -- |
| 35-44 x Year 2015 | -0.13 | (-0.24, -0.02) | 0.016 | -- | -- | -- |
| 45-54 x Year 2015 | -0.16 | (-0.27, -0.05) | 0.003 | -- | -- | -- |
| 55-64 x Year 2015 | -0.22 | (-0.34, -0.11) | <0.001 | -- | -- | -- |
| 65+ x Year 2015 | -0.20 | (-0.33, -0.08) | 0.001 | -- | -- | -- |
| *No. of symptoms^c^* |  |  |  |  |  |  |
| none | *reference* |  |  |  |  |  |
| 1-3 symptoms | -0.17 | (-0.28, 0.06) | 0.002 | -- | -- | -- |
| 4+ more symptoms | -0.40 | (-0.51, -0.30) | <0.001 | -- | -- | -- |
| *Time since diagnosis* | -- | -- | -- | -0.001 | (0.002, 0.0003) | 0.011 |
| *Meals out AD^c^* |  |  |  |  |  |  |
| The same | -- | -- | -- | *reference* |  |  |
| More likely | -- | -- | -- | -0.12 | (-0.24, 0.01) | 0.064 |
| Less likely | -- | -- | -- | -0.07 | (-0.11, -0.04) | <0.001 |
| *Meals out* x *Year 2015* |  |  |  |  |  |  |
| The same x Year 2015 | -- | -- | -- | *reference* |  |  |
| More likely x Year 2015 | -- | -- | -- | 0.05 | (-0.11, 0.21) | 0.515 |
| Less likely x Year 2015 | -- | -- | -- | 0.05 | (0.001, 0.10) | 0.044 |
| *Travel patterns AD^d^* |  |  |  |  |  |  |
| The same | -- | -- | -- | *reference* |  |  |
| More likely to travel | -- | -- | -- | 0.02 | (-0.08, 0.11) | 0.733 |
| Less likely to travel | -- | -- | -- | -0.06 | (-0.10, -0.02) | 0.002 |
| *Travel patterns* x *Year 2015* |  |  |  |  |  |  |
| The same x Year 2015 | -- | -- | -- | *reference* |  |  |
| More likely x Year 2015 | -- | -- | -- | -0.11 | (-0.23, 0.01) | 0.078 |
| Less likely x Year 2015 | -- | -- | -- | -0.01 | (-0.06, 0.04) | 0.634 |

^a^ Sample size: 1938; ^b^ Sample size: 1881; *^c^*Including CD-associated medical conditions; *^d^*AD: after diagnosis
