# Supplementary material for: The impact of diagnosis on health-related quality of life in people with coeliac disease: a UK population-based longitudinal perspective
Source: BMC Gastroenterol. 2019 May 2;19:68. doi: 10.1186/s12876-019-0980-6 (PMC6498641; doi:10.1186/s12876-019-0980-6)
Supplement: Supplementary file 6 — Table S5. Factors associated with quality of life before and after diagnosis – univariate analysis (2015 survey). (DOCX 16 kb) [file 12876_2019_980_MOESM6_ESM.docx]

**Additional file 6**

**Table S5 – Factors associated with quality of life before and after diagnosis – univariate analysis (2015 survey)**

|  | **Before diagnosis** | | | **After diagnosis** | | |
| --- | --- | --- | --- | --- | --- | --- |
| ***Covariates*** | **Coeff.** | **95% CI** | **p-value** | **Coeff.** | **95% CI** | **p-value** |
| *Male* | 0.09 | (0.05, 0.13) | <0.001 | 0.06 | (0.03, 0.08) | <0.001 |
| *Age at diagnosis* |  |  |  |  |  |  |
| less than 18 | *reference* |  |  | *reference* |  |  |
| 18-34 | -0.005 | (-0.07, 0.07) | 0.898 | -0.02 | (-0.07, 0.03) | 0.418 |
| 35-44 | 0.02 | (-0.05, 0.09) | 0.600 | -0.03 | (-0.07, 0.02) | 0.223 |
| 45-54 | 0.04 | (-0.02, 0.11) | 0.190 | -0.02 | (-0.07, 0.02) | 0.341 |
| 55-64 | 0.08 | (0.01, 0.15) | 0.020 | -0.04 | (-0.09, 0.003) | 0.069 |
| 65+ | 0.17 | (0.10, 0.24) | <0.001 | -0.03 | (-0.08, 0.01) | 0.177 |
| *No. of symptoms^a^* |  |  |  |  |  |  |
| none | *reference* |  |  | *reference* |  |  |
| 1-3 symptoms | -0.07 | (-0.11, -0.02) | 0.004 | -0.09 | (-0.12, -0.06) | <0.001 |
| 4+ more symptoms | -0.18 | (-0.23, -0.13) | <0.001 | -0.25 | (-0.28, -0.22) | <0.001 |
| *Max symptoms duration* | -0.003 | (-0.004, -0.002) | <0.001 | -0.008 | (-0.009, -0.006) | <0.001 |
| *No. of comorbidities^b^* |  |  |  |  |  |  |
| None | *reference* |  |  | *reference* |  |  |
| 1 comorbidity | 0.02 | (-0.02, 0.06) | 0.377 | -0.05 | (-0.07, -0.02) | 0.001 |
| 2 comorbidities | -0.02 | (-0.07, 0.04) | 0.527 | -0.08 | (-0.11, -0.04) | <0.001 |
| 3+ comorbidities | -0.07 | (-0.12, -0.01) | 0.023 | -0.21 | (-0.25, 0.17) | <0.001 |
| *Income^a^ <£20,000* | -0.07 | (-0.11, -0.03) | 0.001 | -0.11 | (-0.14, -0.09) | <0.001 |
| *Adherence to a GF diet* |  |  |  |  |  |  |
| All of the time | N/A | N/A | N/A | *reference* |  |  |
| Most of the time | N/A | N/A | N/A | -0.05 | (-0.09, -0.005) | 0.029 |
| Some/little/none of the time | N/A | N/A | N/A | -0.18 | (-0.31, -0.05) | 0.008 |
| *Access to GF products in shops* |  |  |  |  |  |  |
| Very easily |  |  |  | *reference* |  |  |
| Fairly easily | N/A | N/A | N/A | -0.04 | (-0.06, -0.01) | 0.005 |
| Not easily | N/A | N/A | N/A | -0.12 | (-0.17, -0.08) | <0.001 |
| *Meals out* |  |  |  |  |  |  |
| The same | N/A | N/A | N/A | *reference* |  |  |
| More likely | N/A | N/A | N/A | -0.08 | (-0.16, 0.01) | 0.089 |
| Less likely | N/A | N/A | N/A | -0.04 | (-0.07, -0.02) | 0.001 |
| *Travel patterns AD* |  |  |  |  |  |  |
| The same | N/A | N/A | N/A | *reference* |  |  |
| More likely to travel | N/A | N/A | N/A | -0.11 | (-0.19, -0.03) | 0.005 |
| Less likely to travel | N/A | N/A | N/A | -0.09 | (-0.12, -0.07) | <0.001 |
| *Region^a^* |  |  |  |  |  |  |
| North East | -0.05 | (-0.16, 0.07) | 0.426 | -0.05 | (-0.13, 0.02) | 0.173 |
| North West | 0.002 | (-0.07, 0.08) | 0.959 | -0.03 | (-0.08, 0.02) | 0.196 |
| Yorkshire and The Humber | -0.02 | (-0.09, 0.06) | 0.620 | -0.03 | (-0.08, 0.02) | 0.179 |
| East Midlands | -0.03 | (-0.10, 0.05) | 0.470 | -0.04 | (-0.08, 0.01) | 0.164 |
| West Midlands | -0.06 | (-0.15, 0.02) | 0.133 | -0.06 | (-0.12, -0.01) | 0.029 |
| East | -0.02 | (-0.10, 0.05) | 0.557 | -0.03 | (-0.08, 0.02) | 0.214 |
| London | -0.10 | (-0.20, -0.01) | 0.037 | -0.02 | (-0.08, 0.05) | 0.580 |
| South East | *reference* |  |  | *reference* |  |  |
| South West | -0.03 | (-0.10, 0.05) | 0.505 | -0.07 | (-0.11, -0.02) | 0.009 |
| Northern Ireland | -0.06 | (-0.17, 0.06) | 0.341 | -0.05 | (-0.13, 0.03) | 0.206 |
| Scotland | -0.05 | (-0.12, 0.03) | 0.238 | -0.09 | (-0.14, -0.04) | 0.001 |
| Wales | -0.01 | (-0.11, 0.08) | 0.828 | -0.04 | (-0.10, 0.03) | 0.261 |

*^a^*Including CD-associated medical conditions; *^b^*The comorbidity, income, and geographical variables refer to the time of the survey and, therefore, they can only be considered as a proxy for comorbidities, socioeconomic status and geographical location before diagnosis.
