# Supplementary material for: The impact of diagnosis on health-related quality of life in people with coeliac disease: a UK population-based longitudinal perspective
Source: BMC Gastroenterol. 2019 May 2;19:68. doi: 10.1186/s12876-019-0980-6 (PMC6498641; doi:10.1186/s12876-019-0980-6)
Supplement: Supplementary file 7 — Table S6. Additional analysis on 2015 key outcome variables. (DOCX 12 kb) [file 12876_2019_980_MOESM7_ESM.docx]

**Additional file 7**

**Table S6 – Additional analysis on 2015 key outcome variables**

| **Sample stratified by type of medical diagnosis** | **Sample size** | **Duration of symptoms***^a^*  **before diagnosis** | **Self-reported health on EQ-5D tariff –**  **before diagnosis** | **Self-reported health on EQ-5D tariff –**  **after diagnosis** |
| --- | --- | --- | --- | --- |
|  | ***N*** | **Mean (SD)** | **Mean (SD)** | **Mean (SD)** |
| ‘Biopsy only’ | *713* | 12.65 (14.92) | 0.644 (0.334) | 0.845 (0.231) |
| ‘Biopsy and blood test’ | *436* | 12.99 (15.41) | 0.673 (0.315) | 0.857 (0.192) |
| ‘Biopsy only’ +  ‘Biopsy and blood test’ | *1149* | 12.78 (15.10) | 0.655 (0.327) | 0.849 (0.218) |
| ‘Biopsy only’ +  ‘Biopsy and blood test’ +  ‘Blood test only’ | *1298* | 12.75 (15.30) | 0.650 (0.332) | 0.850 (0.217) |
| ‘Biopsy only’ +  ‘Biopsy and blood test’ +  ‘Blood test only’ +  ‘Other medical diagnostic’ | *1319* | 12.72 (15.31) | 0.651 (0.331) | 0.850 (0.217) |
| ‘Biopsy only’ +  ‘Biopsy and blood test’ +  ‘Blood test only’ +  ‘Other medical diagnostic’ +  ‘Medical professional, diagnostic not specified’ | *1336* | 12.76 (15.30) | 0.651 (0.332) | 0.850 (0.219) |

*^a^*Including CD-associated medical conditions
