# Supplementary material for: The impact of diagnosis on health-related quality of life in people with coeliac disease: a UK population-based longitudinal perspective
Source: BMC Gastroenterol. 2019 May 2;19:68. doi: 10.1186/s12876-019-0980-6 (PMC6498641; doi:10.1186/s12876-019-0980-6)
Supplement: Supplementary file 8 — Table S7. Additional analysis on 2015 key outcome variables on the sub-group of individuals aged less than 18 years. (DOCX 12 kb) [file 12876_2019_980_MOESM8_ESM.docx]

**Additional file 8**

**Table S7 -** **Additional analysis on 2015 key outcome variables on the sub-group of individuals aged less than 18 years**

|  | **2015** | | **2006** | | **2015 - 2006** | | |
| --- | --- | --- | --- | --- | --- | --- | --- |
|  | **Mean** | **95% CI** | **Mean** | **95% CI** | **Mean difference** | **95% CI** | **p-value** |
| **Duration (years) of symptoms/associated medical condition pre-diagnosis** | 3.34 | (2.76, 3.92) | 2.78 | (1.94, 3.62) | 0.56 | (-0.45, 1.58) | 0.274 |
|  |  |  |  |  |  |  |  |
| **EQ-5D tariff** |  |  |  |  |  |  |  |
| Pre-diagnosis | 0.57 | (0.51, 0.64) | 0.32 | (0.22, 0.43) | 0.25^***^ | (0.14, 0.37) | <0.001 |
| Time of survey | 0.88 | (0.85, 0.92) | 0.85 | (0.80, 0.91) | 0.03 | (-0.03, 0.08) | 0.378 |
| Change | 0.31^***^ | (0.24, 0.37) | 0.53^***^ | (0.42, 0.64) | -0.22^***^ | (-0.34, -0.11) | <0.001 |
|  |  |  |  |  |  |  |  |
| **Visual Analogue Scale** |  |  |  |  |  |  |  |
| Pre-diagnosis | 46% | (42, 50) | 32% | (26, 37) | 14%^***^ | (8, 20) | <0.001 |
| Time of survey | 83% | (81, 86) | 85% | (82, 89) | -2% | (-6, 2) | 0.385 |
| Change | 37%^***^ | (33, 42) | 53%^***^ | (48, 60) | -16%^***^ | (-23, -8) | <0.001 |

^***^ significant at the 1% level; ^**^significant at the 5% level; ^*^significant at the 10% level
